# In Vitro Evaluation of DSPE-PEG (5000) Amine SWCNT Toxicity and Efficacy as a Novel Nanovector Candidate in Photothermal Therapy by Response Surface Methodology (RSM)

**DOI:** 10.3390/cells10112874

**Published:** 2021-10-25

**Authors:** Naghmeh Hadidi, Niloufar Shahbahrami Moghadam, Gholamreza Pazuki, Parviz Parvin, Fatemeh Shahi

**Affiliations:** 1 Department of Clinical Research and EM Microscope, Pasteur Institute of Iran (PII), Tehran 1316943551, Iran; 2 Department of Nanotechnology, Faculty of New Science and Technology, Tehran Medical Sciences, Islamic Azad University, Tehran 1916893813, Iran; nilou.sh.m@gmail.com; 3 Department of Chemical Engineering, Amirkabir University of Technology, Tehran 158754413, Iran; ghpazuki@aut.ac.ir; 4 Department of Energy Engineering and Physics, Amirkabir University of Technology, Tehran 158754413, Iran; parvin@aut.ac.ir (P.P.); f.shahi.3035@aut.ac.ir (F.S.)

**Keywords:** SWCNT, toxicity, photothermal therapy, DSPE-PEG (5000) amine, response surface methodology (RSM), SKOV3, experimental design

## Abstract

Nowadays, finding a novel, effective, biocompatible, and minimally invasive cancer treatment is of great importance. One of the most promising research fields is the development of biocompatible photothermal nanocarriers. PTT (photothermal therapy) with an NIR (near-infrared) wavelength range (700–2000 nm) would cause cell death by increasing intercellular and intracellular temperature. PTT could also be helpful to overcome drug resistance during cancer treatments. In this study, an amine derivative of phospholipid poly ethylene glycol (DSPE-PEG (5000) amine) was conjugated with SWCNTs (single-walled carbon nanotubes) to reduce their intrinsic toxicity. Toxicity studies were performed on lung, liver, and ovarian cancer cell lines that were reported to show some degree of drug resistance to cisplatin. Toxicity results suggested that DSPE-PEG (5000) amine SWCNTs might be biocompatible photothermal nanocarriers in PTT. Therefore, our next step was to investigate the effect of DSPE-PEG (5000) amine SWCNT concentration, cell treatment time, and laser fluence on the apoptosis/necrosis of SKOV3 cells post-NIR exposure by RSM and experimental design software. It was concluded that photothermal efficacy and total apoptosis would be dose-dependent in terms of DSPE-PEG (5000) amine SWCNT concentration and fluence. Optimal solutions which showed the highest apoptosis and lowest necrosis were then achieved.

## 1. Introduction

Nanomaterials are at the cutting edge of soaring progress in the field of nanotechnology. Nanomaterials boost the biomedical application of new generations of pharmaceutical products, imaging contrast, photosensitizers, or combination therapies in various types of cancer [1,2,3,4]. The application of carbon nanotubes (CNTs) in nanomedicine is a promising perspective. CNTs are nanostructures with distinctive features, including a high surface-area-to-volume ratio, a high loading capacity for drug delivery, and optimal properties as well as thermal conductivity. However, CNTs’ toxicity is still a drawback in regard to their biomedical administration. Covalent and non-covalent functionalization with polymers such as phospholipid PEG derivatives and surfactants would remarkably improve CNTs’ biocompatibility. Moreover, functionalization would also facilitate secondary conjugation with biologicals and drug molecules. Functionalized CNTs are considered as prospective nanocarriers in cancer diagnosis and treatment [5,6].

Photothermal therapy (PTT) is a novel physicochemical therapy for cancers. Optical radiation in the NIR wavelength range of 700–1000 nm in combination with photothermal carriers are the main tools applied in PTT [7]. A suitable and biocompatible photothermal carrier should accumulate, preferably, in cancer cells in comparison to normal cells. When cells are exposed to NIR radiation, photons are absorbed by intercellular and intracellular areas and their energy is converted into heat. As a result, the local temperature increases and this leads to cell death [8]. Tissue transparency in this NIR wavelength (700–1000 nm) is ideal for optical imaging and photothermal therapy. It is reported that CNTs exhibit strong optical absorption in this NIR region. When CNTs are exposed to NIR light, they absorb NIR light, generate heat, and induce the thermal destruction of cancer cells containing significant concentrations of CNTs [9].

Photothermal therapy shows fewer side effects, an easier implementation, and better access to the tumor bed. Therefore, it might be considered as a substitutive treatment protocol in cancers where surgery is not applicable [10,11,12]. Currently, several sources of activating energy such as laser-generated focused ultrasound (FUS) and microwaves are used in photothermal therapy. According to the literature, cancer therapy via laser-induced heat was found to be minimally invasive [13,14,15]. This treatment aims to maximize the absorption of light in the tumor area to generate the required heat for tumor depletion or overcome cellular drug resistance through the inhibition of DNA repair, denaturation of proteins, or disturbing signal transduction [16].

Photothermal nanocarriers that accumulate more in tumor cells and show stronger light absorption in the NIR spectrum are preferable [17,18]. Platinum nanoparticles are photothermal nanocarriers which cause a 70% increase in cell death rate when confronting A2 cancer cells at 1064 nm NIR laser light exposure [19]. Furthermore, polyproline nanostructures [20], molybdenum oxide nanoparticles [21], titanium-containing nanoparticles [8], and copper-made nanostructures were found to cause a significant increase in cancer cell death due to the synergistic effect of these two features. Different laser wavelengths and intensities were also found to be synergistic for reducing cell viability [7,22,23,24]. Gold nanostructures are widely utilized in PTT with various lasers features for different cancer cells [25,26]. The results of these studies also confirmed the synergistic effect of higher intercellular accumulation and NIR absorption rate with cell death. Moreover, a direct relationship was found between cell survival rate, photothermal nanocarrier concentration, laser fluence, and subsequent increase in temperature [27,28,29,30].

Researchers claim that CNTs are thermodynamically favorable to minimize surface tension. CNTs efficiently cross cell membranes due to their nanoneedle cylindrical structures, mechanical flexibility, and high length-to-diameter ratio, and they accumulate in intracellular compartments [31]. When cells with internalized CNTs are exposed to NIR light, they release significant heat and cause the thermal destruction of cells. This local hyperthermia may impede the over-expression of MRP1 (multidrug-resistance-associated protein 1); therefore, drug-resistant cancer cells would become more sensitive to chemotherapeutic drugs [32,33]. The required concentration for carbon nanotubes to achieve similar efficacy is one-tenth in comparison to gold nanorods [34]. Therefore, carbon nanotubes are a better option for PTT compared to gold nanorods [9]. Most PTT studies are based on a high laser power density of 1–40 W/cm^2^. High laser power density and temperature could cause the necrosis of normal tissues. With this in mind, a mild temperature increase with a lower laser power density is preferred in PTT [32]. This strategy would also enhance the efficacy of systemic chemotherapy, especially in MDR (multi-drug-resistant) cancer types [33]. The threshold time of cell necrosis is temperature-dependent. This being the case, it is important to measure the temperature of SWNT-assisted photothermal treatment in order to design a customized PTT protocol for different cancer cells [35,36,37,38,39,40].

In this study, DSPE-PEG (5000) amine (1,2-distearoyl-sn-glycero-3-phosphoethanolamine-*N*-amine poly ethylene glycol) was non-covalently conjugated with SWCNTs to reduce their intrinsic toxicity. Conducting toxicity studies in vital organs, including the lungs and liver, seems essential during the design and development of PTT protocols. Therefore, toxicity studies were performed on lung, liver, and ovarian cancer cell lines that were reported to show some degree of resistance to cisplatin [41,42]. It is also reported that carbon nanotubes themselves can disrupt the cellular respiration cycle, which is the main procedure of MTT. With this in mind, a trypan blue exclusion test was used instead of a pervasive MTT assay to evaluate cell viability [43]. Finally, the photothermal properties (total apoptosis) of DSPE-PEG5000 NH_2_-SWCNTs were investigated in SKOV3 cells, exposed by an NIR laser of 808 nm. The overall correlation and dependence of total apoptosis and necrosis on DSPE-PEG5000 NH_2_-SWCNT concentration, laser fluence (laser power density * laser treatment time), and temperature changes were analyzed simultaneously by RSM and experimental design (Design Expert^®^ version 7) (Version 7.0.0, Stat-Ease, Inc. Minneapolis, MN, USA)to achieve optimal solutions.

## 2. Materials and Methods

### 2.1. Materials

Pure SWCNTs (P2-SWCNTs) were purchased from Carbon Solutions (California, CA, USA). DSPE-PEG (5000) amine was obtained from Nanocs (New York, NY, USA). TNBSA solution (5% *w*/*v* (2,4,6-trinitrobenzene sulfonic acid)) was prepared from Pierce (Rockford, USA). Gold nanorods (GNRs) were prepared from Strem Chemicals (Cambridge, UK). RPMI-1640 medium, McCoy’s 5A medium, and fetal bovine serum (FBS) were purchased from Gibco (New York, NY, USA). Trypsin, penicillin/streptomycin (Pen–Strep), phosphate-buffered saline (PBS), and ethylenediamine tetra acetic acid (EDTA) were obtained from Sigma-Aldrich (Missouri, MO, USA). An apoptosis detection kit (annexin V and 7-AAD (7-aminoactinomycin D)) was ordered from Invitrogen ((California, USA)). HEPG2 (human liver cancer cell line, C158, NCBI), A549 (adenocarcinoma human alveolar basal epithelial cells, C137, NCBI), and SKOV3 (ovarian cancer cell line C209, NCBI) were obtained from the national cell bank of the Pasteur Institute of Iran (Tehran, Iran).

### 2.2. Methods

#### 2.2.1. Preparation and Characterization of DSPE-PEG (5000) Amine SWCNTs

Pure SWCNTs (Carbon Solutions, Riverside, CA, USA) with 4.03 nm of detection efficiency (D50), ≥90% purity carbonaceous material, and 4–7% metal content were purchased. Carbon nanotubes have hydrophobic properties that limit their application in biological systems and biomedicine. DSPE-PEG (5000) amine is a phospholipid derivative of PEG (poly-ethylene-glycol). Non-covalent functionalization was achieved through π-π stacking between the hydrophobic phospholipid chain of DSPE-PEG and the hydrophobic surface of SWCNTs. SWCNTs were first sterilized with dry heat (170 °C for 30 min). One mg of sterile pure SWCNTs were dispersed in about 2 mL of sterile RPMI-1640 medium, and the suspension was sonicated using a probe with 100% amplitude, 120 W power, 20 sec pulse, and 10 s pause for 10 min on ice (UP200Ht, Hielscher, Germany). On the other hand, 8 mg of DSPE-PEG (5000) amine (Nanocs, NY, USA) was added to 2 mL of sterile RPMI-1640 medium and sonicated 3 times in an ultrasonic bath (Sonorex, Bandelin, UK) at 4–6 °C for 15 min. DSPE-PEG solutions were sterilized with 0.22 µm cellulose membrane filters. The synthesis then proceeded under aseptic conditions. The weight ratio of 1-to-8 of DSPE-PEG (5000) amine/SWCNT was selected based on a previous report [36]. Subsequently, the two above suspensions were mixed and sonicated 3 times in an ultrasonic bath at 4–6 °C for 20 min. Then, for homogenization, the suspension was sonicated on ice with an ultrasonic processor using 100% amplitude and 120 W power (UP200Ht Hielsher, Hielscher, Germany) for 2 min (20 s pulse and 10 s pause). To separate non-PEGylated SWCNTs from the supernatant, the suspension was centrifuged at 20,000 rpm (Beckman, IN, USA) at room temperature for 4 h. Finally, the DSPE-PEG (5000) amine/SWCNT concentration in the supernatants was determined by the spectrophotometric method at 280 nm [44,45]. A TNBSA test was performed to determine free amino groups on DSPE-PEG (5000) amine loaded onto SWCNTs [44,45]. Thermogravimetric analysis (TGA) was also performed for pure SWCNTs and DSPE-PEG (5000) amine SWCNTs (functionalized SWCNTs) up to 600 °C. Accordingly, this analysis was performed using a TGA-50 instrument (Shimazu, Tokyo, Japan). The gradual weight loss of DSPE-PEG (5000) amine SWCNTs was measured between 200 °C and 500 °C. By comparing the loss of DSPE-PEG (5000) amine SWCNTs with pure SWCNTs, the amount of PEG loading on SWCNTs was estimated.

Raman spectroscopy with a 532 nm laser and diffusive reflectance method (Nd: YL laser, Almega Thermo Nicolet, Fisher Scientific, Pittsburgh, PA, USA) was applied to characterize the structure of SWCNTs before and after functionalization with DSPE-PEG (5000) amine. The stability of an aqueous dispersion of DSPE-PEG (5000) amine SWCNTs was also studied for 2 months in room temperature. The dispersibility of CNTs can vary from insoluble (sedimented) to soluble (or dispersed). Prepared DSPE-PEG (5000) amine SWCNTs show a black homogeneous dispersion, with no signs of phase separation within 2 months after synthesis (Supplementary Table S1). Atomic force microscopy (AFM) has been utilized for the direct assessment of successful surface functionalization and surface smoothness. EDS (energy-dispersive X-ray spectroscopy) was performed (TESCAN, MIRA II, Czech Republic). XRD (X-ray diffraction) analysis (Philips PW1730, Philips, Eindhoven, Netherlands) was also carried out to study the crystallinity and formation of functionalized SWNTs; the equipment was operated at 30 mA and 40 kV with CuKα radiation (λ = 1.54056 Å). The data were recorded in the 2θ range from 5–80° at a scan rate of 3°/min.

#### 2.2.2. In Vitro Toxicity Studies

In vitro toxicity studies were performed on 3 cell lines, including HEPG2 (C158, NCBI), A549 (C137, NCBI), and SKOV3 (C209, NCBI). HEPG2 and A549 cells were cultured in RPMI 1640 medium that was supplemented with 2 mM L-glutamine, 10% (*v*/*v*) FBS, 100 IU/mL penicillin, and 100 IU/mL streptomycin. The culture media were then kept in a controlled atmosphere (5% CO_2_) and incubated at 37 °C (ASteCC AV114C, UK). The SKOV3 cell line was cultured at 37 °C in a humid atmosphere, containing 5% CO_2_, in McCoy’s culture medium, containing 10% (*v*/*v*) FBS bovine fetal serum and 1% (*v*/*v*) penicillin–streptomycin (100 IU/mL of penicillin and 0.1 mg/mL of streptomycin) in 25 mL flasks. When the cell density reached 90%, they were passaged and then transferred to new flasks [46]. These experiments were performed on cells in the logarithmic phase of growth in conditions of excellent viability (98%), as assessed by a trypan blue exclusion test. During cell culture, invert microscopy (Nikon Eclipse TE2000-U, New York, NY, USA) evaluated morphology changes before and 24 h after treatment with DSPE-PEG (5000) amine SWCNTs and laser radiation (Supplementary Figure S1).

##### Trypan Blue Exclusion Test

A549, HEPG2, and SKOV3, the selected cell lines, were treated with various concentrations of DSPE-PEG (5000) amine SWCNTs for 24 h. HEPG2 and A549 cells were transferred into 96-well plates with a cell density of 1 × 10^4^ cells/well. SKOV3 cells were transferred into 96-well plates with a cell density of 5 × 10^3^ cells/well. Various concentrations of test materials including pure SWCNTs and DSPE-PEG (5000) amine SWCNTs were prepared in the culture medium in such a way that final concentrations of 0, 25, 50, 100, 200, 400, and 600 μg/mL were obtained in each well. Cells were then incubated for 24 h at 37 °C with test materials in a 5% CO_2_ humidified incubator (Astecc AV114C, UK) [47,48]. Morphological changes were observed by an inverted microscope. A trypan blue exclusion test was used to evaluate the effects of non-functionalized and functionalized SWCNTs on the viability of cells. Cell proliferation and the number of viable cells were measured by counting the cells in a Neubauer hemocytometer 24 and 72 h after exposure to pure and DSPE-PEG (5000) amine SWCNTs [47,48].

#### 2.2.3. Transmission Electron Microscopy (TEM)

TEM analysis was performed to evaluate the uptake of DSPE-PEG (5000) amine SWCNTs (25 µg/mL) by SKOV3. [44]. Twenty-four hours after SKOV3 cells were treated with DSPE-PEG (5000) amine SWCNTs, 2 × 10^5^ cells were fixed overnight with glutaraldehyde 3% (Agar Scientific, UK) in phosphate-buffered saline, pH = 7 (Thermo Scientific pH phosphate-buffered saline (PBS), Waltham, MA, USA). After dehydration and resin embedding, ultrathin sections (80 nm thickness) of SKOV3 were prepared using RMC MT-7000 ultramicrotome, stained with uranyl acetate and lead citrate, and examined at 50 KV by a Zeiss EM900 TEM [44] (Supplementary Figure S2).

#### 2.2.4. In Vitro Photothermal Therapy Test in SKOV3 Cells

##### Experimental Design

In order to find the optimal solutions and prevent futile efforts, the experiment was designed based on three independent variables, including DSPE-PEG (5000) amine SWCNT concentration, cell treatment time with DSPE-PEG (5000) amine SWCNTs, and NIR fluence using Design-Expert^®^ software (Version 7.0.0, Stat-Ease, Inc. Minneapolis, MN, USA). Response surface (central composite) design was selected to investigate the effect of variables and their interactions on selected responses. Variables and their constraints were chosen based on previous works and are described in Table 1 [49,50]. Total apoptosis, Y_1_ (%), temperature changes, Y_2_ (∆T ≈ °C), post-NIR exposure time, Y_3_ (hour), and necrosis, Y_4_ (%), were defined as responses. A suitable polynomial model was selected based on significant terms (P, 0.05), coefficient of variation (CV), multiple correlation coefficient (R^2^), and adjusted multiple correlation coefficient (adjusted R^2^).

##### NIR Exposure

SKOV3 cells were transferred into 48-well plates with a cell density of 1 × 10^5^ cells/well. Various concentrations of DSPE-PEG (5000) amine SWCNTs were applied, and final concentrations of 0, 2.5, 13.75, and 25 μg/mL were obtained in each well [49]. Cells were treated with DSPE-PEG (5000) amine SWCNTs for 18 and 24 h. After this period, the medium was removed from all wells and replaced by fresh medium. Then, cells were irradiated with an 808 nm diode laser at a constant intensity of 2 w/cm^2^ for 1, 2, and 3 min, which caused laser fluences of 120, 240, and 360 J/cm^2^, respectively.

##### Temperature Measurement

The temperature of each well content was measured using a thermometer (Fluke 51 II Handheld Digital Probe Thermometer, Washington, DC, USA) during NIR exposure to cells [49].

##### Cell Viability Assessment

An annexin V apoptosis detection kit (annexin V and 7-AAD) was used to investigate the induction of necrosis or apoptosis in SKOV3 cells by flow cytometry 24 and 48 h post-exposure, according to kit’s protocol. Firstly, 1 mL of trypsin (1×) was added to each well and incubated for 2 to 3 min at 37 °C to detach cells. Secondly, 1 mL of the cultured medium containing 10% FBS was added to each well and the detached cells were centrifuged at 300× *g* for 5 min at 4 °C. Cell sediments were then washed by the binding buffer. In a binding buffer with a volume of 80 μL, 1 × 10^5^ cells were dispersed in each microtube. Thereafter, 5 μL of fluorochrome-conjugated annexin V antibody was added to the cell’s suspension. Then, the cells were exposed to the antibody in the staining solution for 30 to 45 min at 4 °C in darkness. The unbounded antibodies were removed by washing them twice with a flow cytometry buffer. In the next step, 200 μL of buffer and 5 μL of the 7-AAD viability staining solution were added to the cells which were incubated in staining solution for 30 to 45 minutes at 4 °C in darkness. To remove the background color for each control sample, a microtube (without annexin V and 7-AAD) was considered as a row cell. Subsequently, the rate of phosphatidylserine uptake at the cell surface was measured by a flow cytometer (BD FACSCalibur) and the results were finally analyzed using FlowJo version 7.6 software [44].

##### Optimization

Optimization was performed by setting variables’ constraints in order to obtain optimal solutions that are desirable in all responses simultaneously (Table 1). Meanwhile, the desirability of optimized solutions was kept at 1. An overlay contour plot was finally constructed to indicate an optimal combination of DSPE-PEG (5000) amine SWCNT concentration, NIR exposure fluence, and cell treatment time to gain results.

### 2.3. Statistical Analysis

Experiments were analyzed by Design-Expert^®^ software (Version 7.0.0, Stat-Ease, Inc. Minneapolis, MO, USA). Significant model terms were defined by a one-way ANOVA, and a p-value less than 0.05 was considered as significant. Results are described as mean ± SD).

## 3. Results 

### 3.1. Characterization of DSPE-PEG (5000) Amine SWCNTs

Using carbon nanotubes in the treatment of cancer is an important emerging phenomenon [51]. However, it is necessary to promote carbon nanotubes’ biocompatibility in a biological medium [52]. DSPE-PEG is one of the most used polymers with a suitable safety profile, and also improves the aqueous solubility of insoluble materials [45,53]. DSPE-PEG might be either adsorbed or covalently bonded to the surface of SWCNTs. DSPE-PEG (5000) amine was non-covalently attached as a functional phospholipid PEG to SWCNTs. Moreover, the TNBSA method, TGA, Raman spectroscopy, elemental analysis, XRD, and AFM were used to ensure surface functionalization. Raman spectroscopy was performed to investigate if there is any change in carbon nanotubes’ molecular structure before and after PEGylation. The IG/ID ratio was reduced from 9.09 to 6.66 in DSPE-PEG (5000) amine SWCNTs (Supplementary Figure S3). Meanwhile, there was no shift in the D and G bands in the DSPE-PEG (5000) amine SWCNT spectrum, which confirms non-covalent PEGylation. As shown in the supplementary files, the loading of DSPE-PEG onto the final complex was estimated by elemental analysis to be 58 ± 2.5% based on carbon, nitrogen, and hydrogen contents (Supplementary Table S2).

**Figure 1:**
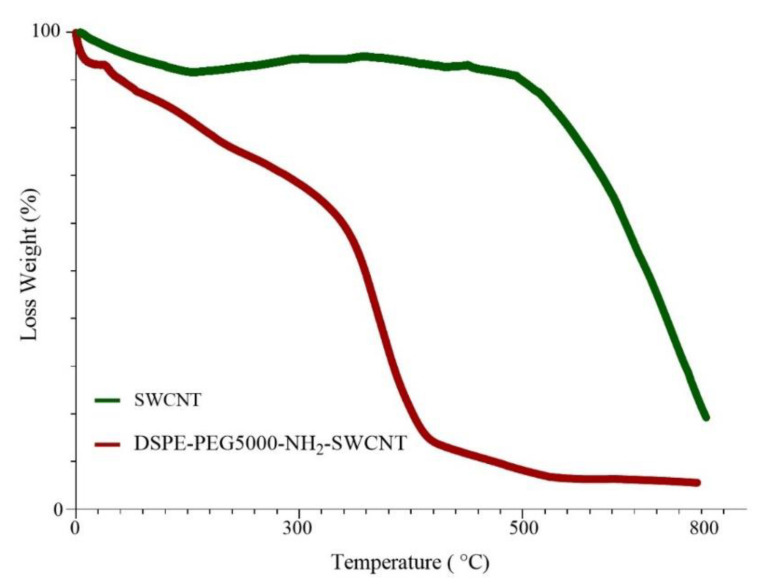
TGA diagram of pure SWCNTs and DSPE-PEG (5000) amine SWCNTs.

A TGA diagram of pure and functionalized carbon nanotubes is shown in Figure 1. Comparing these two graphs, 66.22% weight loss was observed in DSPE-PEG (5000) amine SWCNT samples in the range of 100 to 450 °C. As for pure SWCNTs, a total mass loss of 6.38% is depicted. If we consider the weight loss of the pure sample as a reference, the % loading of DSPE-PEGs would be 59.84% (Figure 1), which is nearly the same as elemental analysis data. DSPE-PEG loading efficiency was about 91% ± 0.5%, as obtained by the TNBS method.

**Figure 2:**
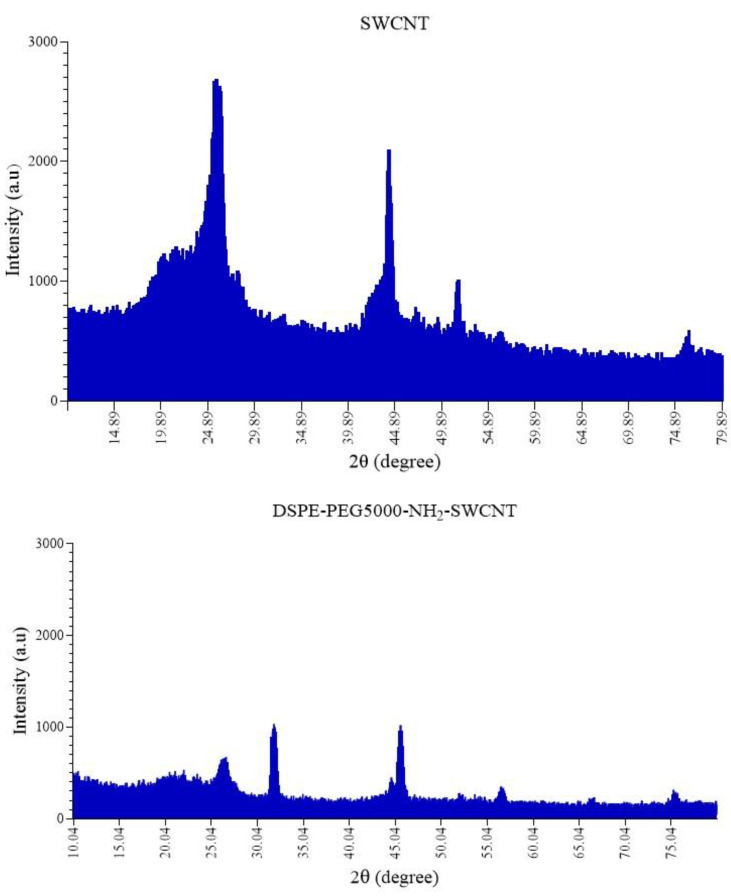
XRD patterns of pure SWCNTs and DSPE-PEG (5000) amine SWCNTs.

The XRD patterns of SWCNTs and DSPE-PEG (5000) amine SWCNTs were presented in Figure 2. XRD analysis is a unique method to find qualitative properties, such as crystallinity, types, fingerprints, and quantitative material. The main peaks of CNTs are observed at ≈25° (002) and 43° (100). The length and width of peaks were commonly referring to a crystalline or amorphous nature; thus, the width increases with reducing intensity or length, which refers to existing in an amorphous crystal form. Typically, impurities and surface polymer conjugation directly affect the position and intensity of the peaks. As illustrated in Figure 2, functionalization has reduced the intensity of peaks. Peak shifts and new peak emergence are other points of evidence that confirm a new, amorphous structure. 

AFM analysis indicates that functionalized SWCNTs are better separated and less aggregated than raw SWCNTs. The average Ra (roughness average), Rq (root mean square (RMS) roughness), and Rt (maximum height of the profile) of SWCNTs and DSPE-PEG (5000) amine SWCNTs are shown in Table 2. More surface smoothness in DSPE-PEG (5000) amine SWCNTs was confirmed by a lower Ra in and RT comparison to SWCNTs.

### 3.2. In Vitro Toxicity Studies

#### Trypan Blue Exclusion Test

The IC_50_ of the three cancerous cell lines was calculated based on trypan blue dye exclusion assays. It was found that DSPE-PEG (5000) amine SWCNTs were substantially less toxic compared to pure SWCNTs. These findings were also reported by our previous studies [44,45,47,48]. It was assumed that the molecular weight of DSPE-PEG and its functional groups played an important role in cell viability. Different cells would have a different IC_50_ when they are treated with DSPE-PEG (5000) amine SWCNTs, but the overall conclusion is that our non-covalent protocol seemed to be effective in increasing SWCNT biocompatibility in different cell lines. The IC_50_ after 24 h in HEPG2, A549, and SKOV3 cells was 300, 370, and 50 µg/mL, respectively (Table 3). PTT studies were performed on SKOV3 cells as a model of cancerous cells.

### 3.3. In Vitro Photothermal Therapy Test in SKOV3 Cells

#### Experimental Design

Experimental runs and responses are summarized in Table 4. Analysis of variance of responses showed that a quadratic model was the most appropriate model fitted to the results (*p*, 0.05). The correlations between variables and statistical parameters were defined in Equations (1)–(4) (e1–4).

Y_1_ (Total apoptosis) = 59 + 10.9 A + 8.71 B − 2.09 C − 0.28 − 0.74 B C + 1.75 A^2^ − 11.33 B^2^
CV (%) = CV (%) = 8.04 R^2^ = 0.90 adjusted R^2^ = 0.86
(1)Y_2_ (Temperature changes (∆T)) = 11.58 + 3 A + 3.78 B − 0.34 C + 1.82 A B − 0.2 A C + 0.55 B C − 1.10 A^2^ − 1.28 B^2^
CV (%) = 6.64 R^2^ = 0.92 adjusted R^2^ = 0.90
(2)Y_3_ (Post NIR exposure time) = 36 + 0 A + 0 B + 0 C
CV (%) = 7.82 R^2^ = 0.81 adjusted R^2^ = 0.75
(3)Y_4_ (Necrosis) = 5.87 + 1.07 A + 0.8 B – 1.47 C + 0.42 A B + 0.029 A C – 0.91 B C + 0.4 A^2^ – 0.22 B^2^
CV (%) = 6.12 R^2^ = 0.89 adjusted R^2^ = 0.77
(4)

**Figure 3:**
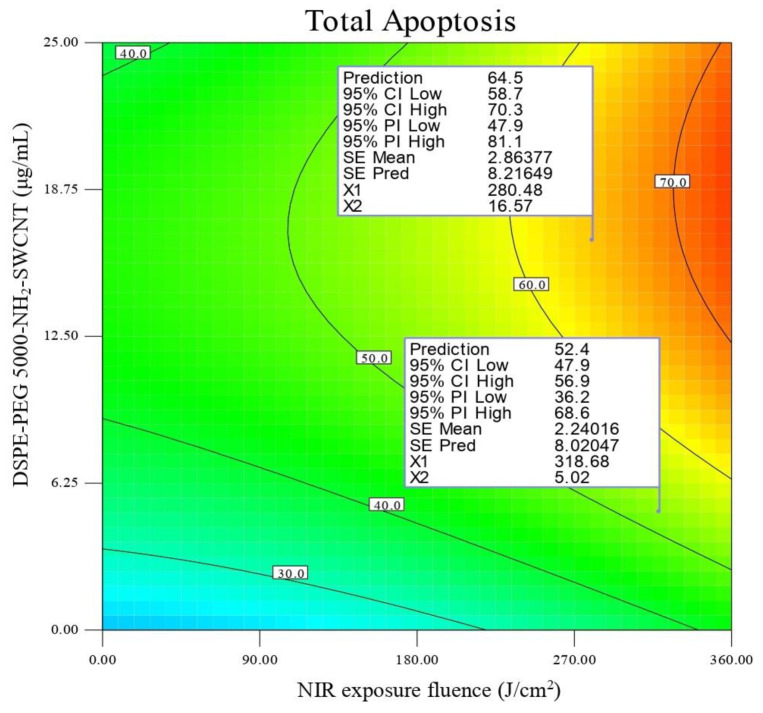
Contour plot of total apoptosis (Y1) in SKOV3 cells.

From Y1, it was concluded that the model terms A and B have a significant positive effect, but that B^2^ has a significant negative effect on total apoptosis. From Y_2_ it was concluded that model terms A, B, and AB positively affect temperature changes (∆T), whereas model terms A^2^ and B^2^ had negative effects on Y_2_. As mentioned above, none of the model terms significantly positively or negatively affect Y_3_. As shown in Equation (4) (e4), necrosis (Y_4_) would be affected positively by model term A, while the reverse was observed for model terms C and BC. The response surface contour for total apoptosis (Y_1_) is shown in Figure 3. This contour plot suggests that a total apoptosis of ≥65% would be achieved in DSPE-PEG (5000) amine SWNT concentrations of 12.5–25 µg/mL and an NIR exposure fluence of 310-360 J/cm^2^.

**Figure 4:**
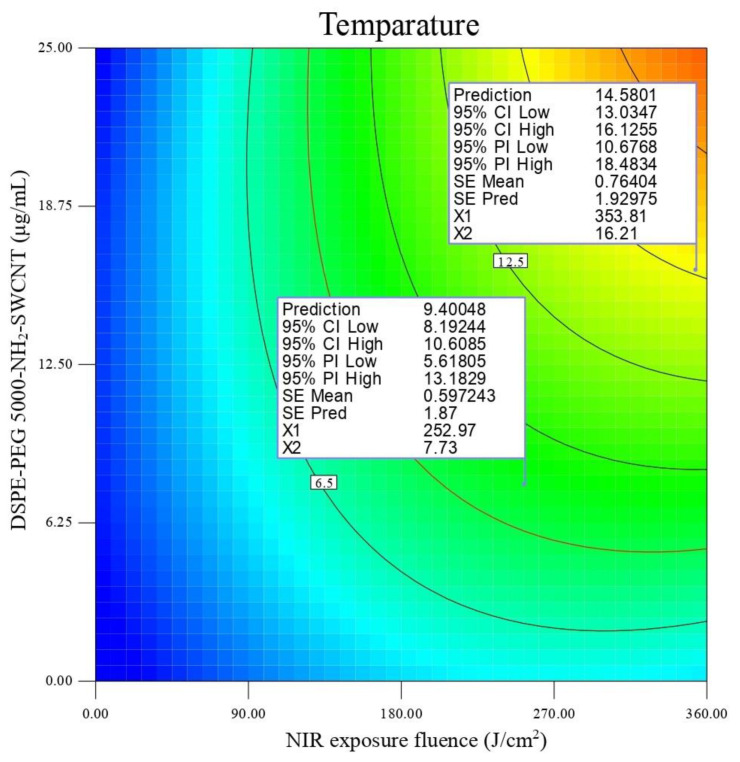
Contour plot of temperature changes (∆T ≈ °C) (Y2) in SKOV3 cells.

As is observed in Figure 4, NIR exposure fluence and DSPE-PEG (5000) amine SWNT concentration would also directly cause temperature changes in experimental runs. These findings are in accordance with similar studies [54,55]. Our study suggests that temperature changes (∆T) of 9.2–16 °C (Y_2_) would be achieved in DSPE-PEG (5000) amine SWNT concentrations of 12.5–25 µg/mLy and an NIR exposure fluence of 310–360 J/cm^2^. These temperature changes would cause a total apoptosis of ≥65% in SKOV3 cells.

**Figure 5:**
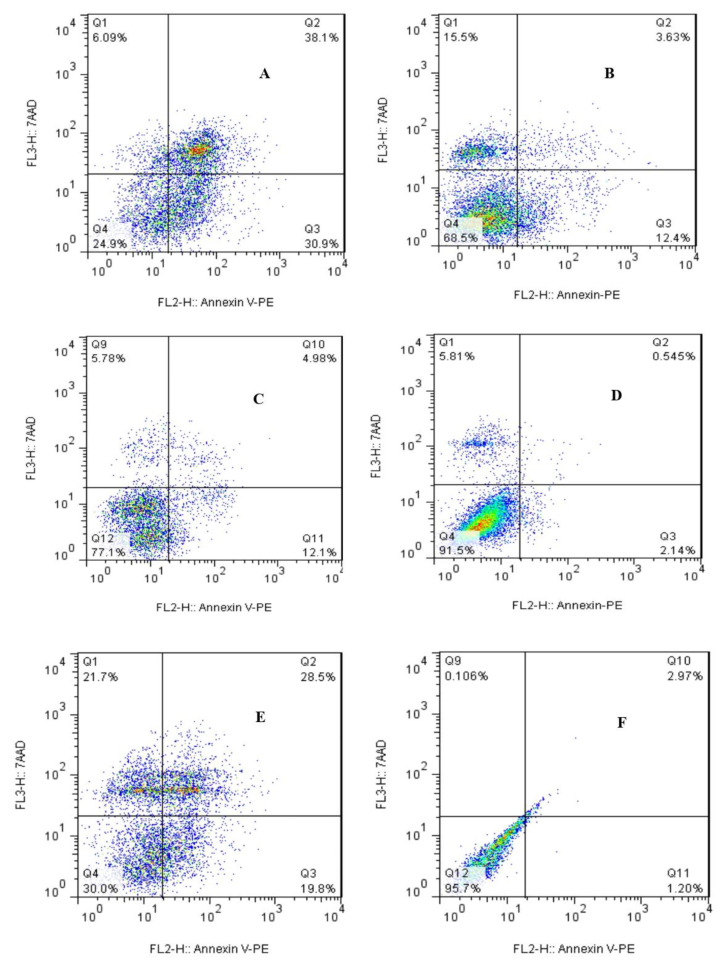
Flow cytometry analysis of SKOV3 cells, 24 h post-NIR exposure: (**A**) DSPE-PEG (5000) amine SWCNTs (25 μg/mL) with NIR exposure fluence (360 J/cm^2^), (**B**) DSPE-PEG (5000) amine SWCNTs (0 μg/mL) with NIR exposure fluence (360 J/cm^2^), (**C**) DSPE-PEG (5000) amine SWCNTs (25 μg/mL) without NIR exposure, (**D**) DSPE-PEG (5000) amine SWCNTs (0 μg/mL) without NIR exposure fluence (negative control), (**E**) GNRs (25 μg/mL) with NIR exposure fluence (360 J/cm^2^) (positive control), and (**F**) unstained sample.

Photothermal therapy (PTT) causes cancer cell death via apoptosis and/or necrosis [56,57,58]. All DSPE-PEG (5000) amine SWCNT concentrations are below IC_50_. As shown in Figure 5, cell treatment times had no significant effect on early and late apoptosis; however, a moderate effect on late apoptosis was observed in the absence of NIR exposure. The effect of NIR exposure on cell viability was investigated after laser fluences of 120, 240, and 360 J/cm^2^. The results were in accordance with other research which proposed a maximum of 3 min exposure or a laser fluence of 360 J/cm^2^ to achieve the best results [59,60]. GNRs (25 μg /mL) with an NIR exposure fluence of 360 J/cm^2^ have been used as a positive control group [61]. Annexin-V-staining-positive cells were found to be associated with early apoptosis, 7-AAD-staining-positive cells were associated with necrosis, and annexin-V- 7-AAD-staining-positive cells were found to be associated with late apoptosis. Necrosis was considered not significant as it did not exceed 12.5% in experimental runs. Therefore, the possibility of necrosis was ruled out and apoptosis was confirmed as the main mechanism of cell death (Figure 5).

### 3.4. Optimization

**Figure 6:**
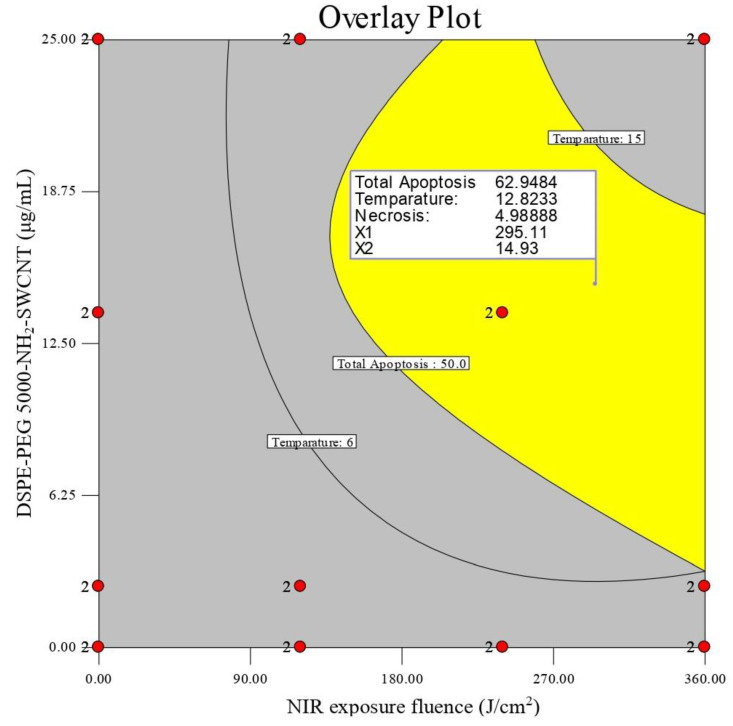
Overlay plot of optimal results (Red points show some of the experiments included in design algorithm).

Optimization was performed according to constraints defined in Table 1. An overlay contour plot was constructed (Figure 6). Optimal solutions with a desirability of ≥81% for all four responses were listed in supplementary Table S3.

As is shown in Figure 6, minimal necrosis (≤5%), maximum total apoptosis (52.0–64.5%), and temperature changes (∆T 9.5–14.5 °C) could be achieved at an NIR exposure fluence in the range of 263–345 J/cm^2^ and with DSPE-PEG (5000) amine SWCNTs at 14.5–20.5 μg/mL. Moreover, cell treatment time did not have any significant effect on responses, and was constant during optimization. 

## 4. Discussion

Carbon nanotubes (CNTs) possess excellent characteristics, including strong optical absorption in the near-infrared region (NIR) and photoluminescence as well as photoacoustic properties, which are helpful for tracking CNTs in biological systems. CNTs would offer an efficient platform for biomedical applications due to their high surface ratio. Despite an ever-growing body of evidence indicating that nanomaterials are the next generation of biological and pharmaceutical products, the polemical statements of skeptics point toward their cytotoxicity, which poses a threat to their pervasive global consumption. In the same line, the main disadvantages of CNTs are their poor solubility and high toxicity, which would be considered a great milestone in their biomedical application. Functionalization with suitable polymers would facilitate their application in biomedicine by improving their solubility, biocompatibility, and more targeted cell internalization [44,45,60,61]. The physical properties of CNTs, preferably SWCNTs, such as greater NIR absorbance, are responsible for their photoacoustic imaging capabilities [55]. Considering the high surface area of SWCNTs, they could be suggested as ultrasensitive biosensors and nanocarriers for drug delivery [44,45,61]. Th excellent photothermal conversion efficiency of SWCNTs might be used in upcoming cancer guidelines. However, the biocompatibility of SWCNTs would be very critical for their biomedical application, and must be addressed in the first place [45,47,62]. It was stated that suitable polymer conjugation and/or functionalization would improve CNTs’ aqueous solubility and biocompatibility. Functionalization might also facilitate tumor cells’ internalization, and more selective cytotoxicity might be achieved [61].

Photothermal therapy (PTT) causes tumor destruction via the apoptosis and/or necrosis of cancerous cells [56,57,58]. Apoptosis is an internal process that is considered the best method of killing cells, due to little to no extracellular leakage or no subsequent inflammation [57]. In contrast to necrosis, apoptosis results in apoptotic bodies that can be phagocytosed by antigen-presenting cells (APCs). This phenomenon would prevent tumor growth [58]. Cells are more sensitive to heat during their S phase due to hyperthermia’s destructive effect on proteins [63]. Many studies report that mild hyperthermia (42–45 °C) is optimal to induce apoptosis via mitochondrial damage and oxidative stress. Higher temperatures (≥52 °C) could damage normal cells and increase PTT adverse effects [64,65]. As a matter of fact, cell necrosis is temperature-dependent. This being the case, it is important to customize cells’ temperature changes in SWNT-assisted photothermal therapy. Local hyperthermia might also impede the over-expression of MRP1 (multidrug-resistance-associated protein 1) and the vascular permeability of tumor cells. As a result, the delivery of anticancer drugs into tumors would be facilitated and drug-resistant cancer cells would become more sensitive to conventional chemotherapies [32,33]. A mild photothermal therapy plan with low NIR exposure fluence and temperature changes <15 °C would be preferable in cancer treatment [32].

In this study, we synthesized DSPE-PEG (5000) amine SWCNTs as a novel, suitable, and biocompatible photothermal nanocarrier for PTT of ovarian cancer (SKOV3 cells). DSPE-PEG (5000) amine was non-covalently attached to SWCNTs with loading of 58% and a loading efficiency of 91% in order to improve their aqueous dispersibility and biocompatibility. We performed cell viability studies by using a trypan blue exclusion test and an apoptosis kit for flow cytometry analysis; by doing so, the overall interaction of and nanomaterials’ impact on cells from a toxological perspective would be somehow explainable for the further contribution of these nanomaterials in PTT of various cancers [62]. A loss of glutathione, a distinguished change in cellular thiol levels, cytoplasmic reactive oxygen species (ROS), and mitochondrial superoxide are major factors affecting oxidative damage, which may underlie cell apoptosis in cells exposed to SWCNTs. However, surface modifications in functionalized SWCNTs could modulate cellular death mechanisms. The application of biocompatible photothermal nanocarriers would minimize adverse effects of PTT. Acknowledging this, we first examined the in vitro toxicity of DSPE-PEG (5000) amine SWCNTs in vital organs including the liver and lungs. It is necessary to check if the concentrations of these nanomaterials are safe to be used in future biomedical applications or not [61,62,65,66]. The molecular weight and functional group charges of DSPE-PEG were assumed to play an important role in the biocompatibility improvement of DSPE-PEG amine SWCNTs [45,47,48]. More hydrophobicity and positive charges of amine groups in DSPE-PEG (5000) amine, was assumed to potentiate cell internalization in comparison to DSPE-PEG 2000 and DSPE-PEG COOH.

DSPE-PEG (5000) amine SWCNTs were shown to be biocompatible in monocyte-derived dendritic cells (MDDCs) and Jurkat cells (T lymphocytes) [44,47]. Our current study indicates that A549, HEPG2, and SKOV3 would have different 24- and 72-hour IC50s after treatment with DSPE-PEG (5000) amine SWCNTs [44,45,47,48]. However, it seems that DSPE-PEG5000 NH2-SWCNTs were more toxic in SKOV3, which was confirmed by an IC50 of 50 µg/mL after 24 h. Therefore, it was suggested to investigate the application of optimim solutions in PTT of ovarian cancer in-vivo. A lower IC_50_ would be somehow explained by the over-expression of negative charges of folate receptors in SKOV3 cells and the better cellular uptake of positively charged amine-functionalized CNTs [45]. Liang et al. also declare that PEGylated SWNT-Cy5.5 could be a promising photothermal-responsive candidate for PTT in folate-receptor-positive breast cancers [38].

## 5. Conclusions

We synthesized functionalized SWCNTs (DSPE-PEG (5000) amine SWCNTs) as a novel, suitable, and biocompatible photothermal nanocarrier for PTT of ovarian cancer (SKOV3 cells) with NIR exposure fluence in vitro. It was shown that minimal necrosis (≤5%), maximum total apoptosis (52.0–64.5%), and optimal temperature changes (∆T 9.5–14.5 °C) could be achieved at an NIR exposure fluence of 263–345 J/cm^2^ and 14.5–20.5 μg/mL of DSPE-PEG (5000) amine SWCNTs. Moreover, cell treatment time did not have any significant effect on responses and was kept constant during optimization. Temperature changes (∆T < 15 °C) are known as the best temperature option to destruct tumors locally. Moreover, the predominant cell death mechanism observed in optimal solutions was apoptosis and no significant necrosis was reported in vitro. However, it is recommended to perform complementary in vivo studies to ensure the efficacy of DSPE-PEG (5000) amine SWCNTs as a novel and biocompatible photothermal nanocarrier for PTT of ovarian cancer.

## Figures and Tables

**Table 1:** Variables for experimental design and response surface methodology (central composite).

**Factor**	**Variables**	**Constraints**	**Optimization Criteria**	**Weight**	**Importance**
A	NIR exposure fluence (J/cm^2^)	120–360	In range	1	3
B	DSPE-PEG 5000-NH_2_-SWCNT (μg/mL)	2.5–25	In range	1	3
C	Cell Treatment Time (hour)	18, 24	24	1	3
Response					
Y_1_	Total Apoptosis (%)	6.2–78.87	Maximize (≥50%)	1	3
Y_2_	Temperature change (∆T ≈ °C)	0–19.75	In range (10–15 °C)	1	3
Y_3_	Post NIR exposure time (h)	24, 48	none	1	3
Y_4_	Necrosis (%)	1.52–12.53	Minimize (≤8%)	1	3

**Table 2:** Roughness analysis of SWCNTs and DSPE-PEG (5000) amine SWCNTs by AFM (*n* = 3).

	Ra (pm)	Rq (pm)	Rt (nm)
SWCNT	1161 ± 7.82	2456 ± 1.29	48.99 ± 4.99
DSPE-PEG 5000-NH_2_-SWCNT	312.35 ± 6.65	455 ± 2.86	7.628 ± 2.23

**Table 3:** Twenty-four and seventy-two hours IC_50_ (µg/mL) of HEPG2, A549, and SKOV3 cells after treatment with pure SWCNTs and DSPE-PEG (5000) amine SWNTs (*n* = 3).

Cell Line	Samples	IC50 (µg/mL) after	
		24 h	72 h
HEPG2	Pure SWCNT DSPE-PEG 5000-NH_2_-SWNTs	150 ± 6.35 300 ± 8.1	50 ± 7.50 250 ± 6.95
A549	Pure SWCNT DSPE-PEG 5000-NH_2_-SWNTs	150 ± 7.6 370 ± 4.5	50 ± 4.95 240 ± 6.78
SKOV3	Pure SWCNT DSPE-PEG 5000-NH_2_-SWNTs	150 ± 7.56 50 ± 6.77	50 ± 4.8 20 ± 2.6

**Table 4:** Experimental runs designed by RSM (central composite design).

Independent Variables				Responses			
Run	A: DSPE-PEG5000-NH_2_-SWCNT Concentration (μg/mL)	B: NIR Exposure Fluence (J/cm^2^)	C: Cell Treatment Time (h)	Y_1_: Total Apoptosis (%)	Y_2_: ∆T (°C)	Y_3_: Post NIR Exposure (h)	Y_4_: Necrosis (%)
1	2.5	120	18	36.65 ± 2.46	4 ± 2.86	24	5.93 ± 3.24
2	2.5	120	18	31.10 ± 2.41	4.4 ± 4.29	48	2.70 ± 1.12
3	2.5	120	24	18.13 ± 1.09	4 ± 1.73	24	7.11 ± 2.60
4	2.5	120	24	38.97 ± 1.31	0.65 ± 0.69	48	2.99 ± 1.06
5	0	120	18	24.92 ± 2.25	5.5 ± 5.05	24	5.08 ± 1.29
6	0	120	18	30.42 ± 2.16	3.6 ± 1.3	48	2.52 ± 1.21
7	0	120	24	20.47 ± 2.98	2.85 ± 1.05	24	6.80 ± 1.64
8	0	120	24	33.18 ± 1.68	2.26 ± 0.33	48	3.06 ± 1.67
9	25	120	18	51.93 ± 3.84	10.3 ± 0.49	24	11.58 ± 4.13
10	25	120	18	42.93 ± 2.14	6.73 ± 3.30	48	4.95 ± 0.17
11	25	120	24	28.44 ± 1.74	10.03 ± 0.46	24	1.52 ± 1.20
12	25	120	24	43.94 ± 1.85	8.9 ± 2.5	48	2.77 ± 1.72
13	2.5	0	18	37.03 ± 1.27	0	24	7.50 ± 0.92
14	2.5	0	18	30.88 ± 1.46	0	48	4.03 ± 1.75
15	2.5	0	24	32.63 ± 1.74	0	24	7.05 ± 3.45
16	2.5	0	24	36.59 ± 5.19	0	48	3.53 ± 1.86
17	25	0	18	44.04 ± 2.33	0	24	7.20 ± 1.97
18	25	0	18	35.02 ± 0.38	0	48	5.08 ± 1.75
19	25	0	24	36.63 ± 2.38	0	24	5.26 ± 1.61
20	25	0	24	46.12 ± 4.15	0	48	3.55 ± 1.16
21	13.75	0	18	50.43 ± 1.55	0	24	4.82 ± 3.58
22	13.75	0	18	38.98 ± 0.94	0	48	10.42 ± 1.38
23	13.75	0	24	37.69 ± 4.37	0	24	2.62 ± 1.89
24	13.75	0	24	46.64 ± 5.97	0	48	2.46 ± 1.02
25	13.75	240	18	61.54 ± 4.49	12.4 ± 1.49	24	7.43 ± 3.42
26	13.75	240	18	59.02 ± 3.27	14.13 ± 1.40	48	4.57 ± 1.79
27	13.75	240	24	46.45 ± 3.48	14.13 ± 2.46	24	6.74 ± 4.74
28	13.75	240	24	63.53 ± 5.36	9.68 ± 1.04	48	4.45 ± 2.41
29	0	240	18	42.30 ± 3.85	3.5 ± 1.30	24	6.43 ± 5.15
30	0	240	18	38.16 ± 2.84	6.43 ± 2.0	48	2.81 ± 0.42
31	0	240	24	22.84 ± 1.17	2.6 ± 0.7	24	8.00 ± 2.10
32	0	240	24	32.84 ± 2.72	3 ± 0.1	48	1.77 ± 0.95
33	2.5	360	18	43.23 ± 4.85	12.1 ± 4.95	24	6.56 ± 2.22
34	2.5	360	18	48.88 ± 3.24	7.42 ± 1.28	48	5.83 ± 3.41
35	2.5	360	24	45.68 ± 2.84	5.95 ± 1.39	24	6.59 ± 0.79
36	2.5	360	24	60.22 ± 3.33	5.74 ± 1.38	48	6.33 ± 1.31
37	0	360	18	37.85 ± 2.46	3.33 ± 1.32	24	5.03 ± 1.81
38	0	360	18	44.25 ± 2.83	6.76 ± 0.49	48	7.58 ± 1.53
39	0	360	24	31.97 ± 3.20	3.36 ± 0.23	24	4.81 ± 1.65
40	0	360	24	46.08 ± 5.17	3.76 ± 0.57	48	2.58 ± 0.80
41	25	360	18	78.87 ± 2.99	19.62 ± 1.8	24	11.46 ± 1.38
42	25	360	18	73.38 ± 2.25	12.87 ± 1.74	48	12.53 ± 3.91
43	25	360	24	61.43 ± 5.93	19.75 ± 1.24	24	3.22 ± 2.15
44	25	360	24	76.64 ± 2.60	16.07 ± 0.65	48	6.25 ± 1.22
45	0	0	18	18.95 ± 4.66	0	24	10.89 ± 4.75
46	0	0	18	8.52 ± 3.02	0	48	4.21 ± 2.13
47	0	0	24	6.20 ± 1.19	0	24	2.47 ± 2.16
48	0	0	24	18.98 ± 4.91	0	48	1.52 ± 1.37

## Data Availability

Not applicable.

## Supplementary Materials

The following are available online at https://www.mdpi.com/article/10.3390/cells10112874/s1, Figure S1: Morphological changes of SKOV3 (A) before and (B) 24 hours after treatment with DSPE-PEG-SWCNT and laser radiation; Figure S2: TEM analysis of uptake of DSPE-PEG5000/SWCNT by SKOV3 (A) magnification 3000, (B) magnification 12000; Figure S3: Raman spectra of (A) pure SWCNTs, (B) DSPE-PEG5000-NH2-SWCNT; Table S1: Stability studies of DSPE-PEG5000-NH2-SWCNTs in room temperature; Table S2: elemental analysis of pure SWCNT and DSPE-PEG5000-NH2-SWCNT; Table S3: Optimum solutions for all 4 responses.
