# Supplementary material for: In Vitro Evaluation of DSPE-PEG (5000) Amine SWCNT Toxicity and Efficacy as a Novel Nanovector Candidate in Photothermal Therapy by Response Surface Methodology (RSM)
Source: Cells. 2021 Oct 25;10(11):2874. doi: 10.3390/cells10112874 (PMC8616160; doi:10.3390/cells10112874)
Supplement: Supplementary file 1 [file cells-10-02874-s001.zip › cells-1420947-supplementary.pdf]

In -vitro Evaluation of DSPE-PEG 5000-NH_2-SWCNT toxicity and efficacy as a novel nano vector candidate in photothermal therapy by Response Surface Methodology (RSM)

Naghmeh Hadidi [1]*, Niloufar Shahbahrami Moghadam [2], Gholamreza Pazuki [3], Parviz Parvin [4], Fatemeh Shahi[4]

1. Department of Clinical Research and EM Microscope, Pasteur Institute of Iran (PII), Tehran, Iran
2. Department of Nanotechnology, Faculty of New Science and Technology, Tehran Medical Sciences, Islamic Azad University, Tehran, Iran
3. Department of Chemical Engineering, Amirkabir University of Technology, Tehran, Iran
4. Department of Energy Engineering and Physics, Amirkabir University of Technology, Tehran, Iran

- *Author for correspondence:

  Naghmeh Hadidi, Tel: +98-21-64112808,+989122054655 ,
  E-mail: n_hadidi@pasteur.ac.ir and hadidi@gmail.com

Supplementary table 1): Stability studies of DSPE-PEG5000-NH_2-SWCNTs in room temperature

	SWCNTs aqueous concentration (mg/ml)_ Initial	SWCNTs aqueous concentration (mg/ml)_ After 2 months	% Decrease in SWCNTs aqueous concentration (mg/ml)_After 2 months
1	0.21	0.20	4.76
2	0.31	0.29	6.45
3	0.58	0.56	3.45
4	0.52	0.50	3.85
5	0.77	0.74	3.90
6	0.12	0.10	0.00
7	0.71	0.67	5.63
8	0.61	0.60	1.64
9	0.90	0.85	5.56
10	0.14	0.12	14.29
11	0.84	0.80	4.76
12	0.81	0.79	2.47
13	0.78	0.75	3.85
14	0.75	0.71	5.33
15	0.82	0.80	2.44

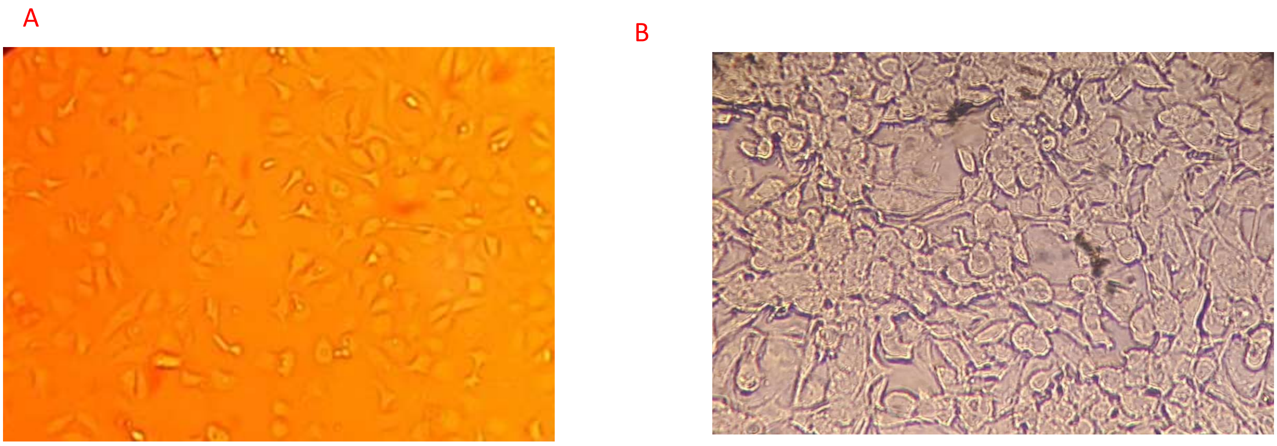

Supplementary Figure 1): Morphological changes of SKOV3 A) before and B) 24 hours after treatment with DSPE-PEG-SWCNT and laser radiation

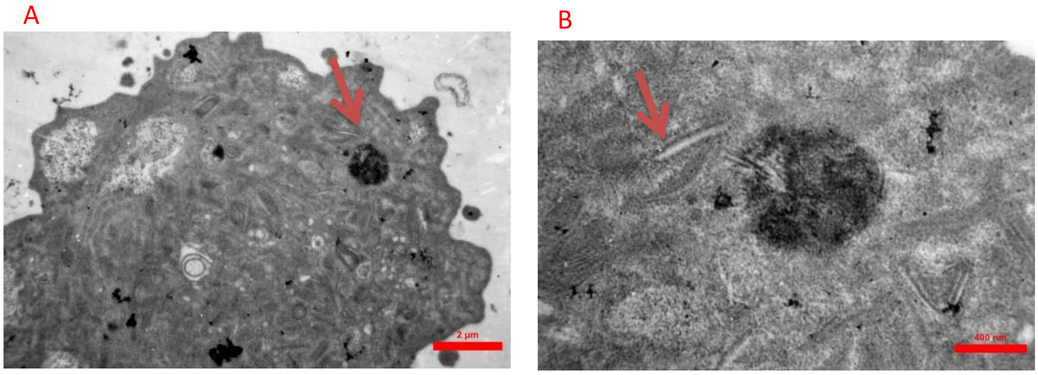

Supplementary Figure 2): TEM analysis of uptake of DSPE-PEG5000/SWCNT by SKOV3 A) magnification 3000, B) magnification 12000

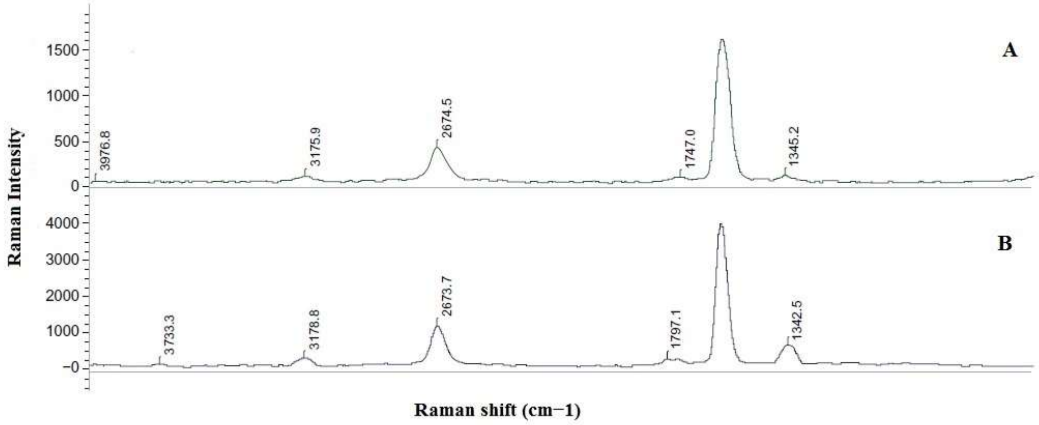

Supplementary Figure 3): Raman spectra of A) pure SWCNTs, B) DSPE-PEG5000-NH_2-SWCNT

Supplementary table 2): elemental analysis of pure SWCNT and DSPE-PEG5000-NH_2-SWCNT

Element	SWCNTs	DSPE-PEG5000-NH_2	DSPE-PEG5000-NH_2-SWCNTs	DSPE-PEG-loading %
N	-	0.7%	2.82%	
C	85%	55.37%	67.60%	58%
H	-	8.72%	3.57%	

% Carbon in DSPE-PEG-SWCNT= (SWCNT portion × 85)+ (DSPE-PEG5000 portion × 55)

Supplementary table 3): Optimum solutions and desirability

	NIR exposure fluence (J/cm^2)	DSPE-PEG-SWCNT concentration (µg/ml)	Total apoptosis (%)	Temparature (°C)	Necrosis (%)	Desirability
1	289.02	18.58	63.2	14.15	4.89	0.810
2	290.12	18.62	63.3	14.19	4.91	0.810
3	289.53	18.51	63.2	14.13	4.90	0.810
4	286.47	18.46	62.9	14.05	4.86	0.810
5	292.90	18.16	63.6	14.08	4.95	0.810
6	275.75	18.22	61.8	13.71	4.73	0.810
7	291.95	17.69	63.5	13.88	4.94	0.810
8	279.76	19.53	62.0	14.25	4.76	0.810
9	269.26	19.06	61.0	13.83	4.63	0.810
10	310.27	19.28	65.4	14.85	5.19	0.809
11	308.94	19.49	65.2	14.90	5.16	0.809
12	354.50	17.95	70.7	14.99	5.88	0.803
13	154.64	16.96	51.2	9.46	3.68	0.789
14	146.52	21.40	48.9	9.78	3.38	0.785
15	176.84	25.00	47.5	11.56	3.37	0.776
